# Seasonal Changes in Photoperiod: Effects on Growth and Redox Signaling Patterns in Atlantic Salmon Postsmolts

**DOI:** 10.3390/antiox12081546

**Published:** 2023-08-02

**Authors:** Peng Yin, Takaya Saito, Per Gunnar Fjelldal, Björn Thrandur Björnsson, Sofie Charlotte Remø, Tom Johnny Hansen, Sandeep Sharma, Rolf Erik Olsen, Kristin Hamre

**Affiliations:** 1 Institute of Marine Research, 5817 Bergen, Norway; peng.yin@hi.no (P.Y.); takaya.saito@hi.no (T.S.); sofie.remo@hi.no (S.C.R.); 2 Department of Biological Sciences, University of Bergen, 5020 Bergen, Norway; 3 Institute of Marine Research, Matre, 5984 Matredal, Norway; pergf@hi.no (P.G.F.); tomh@hi.no (T.J.H.); 4 Department of Biological and Environmental Sciences, University of Gothenburg, 41390 Gothenburg, Sweden; thrandur.bjornsson@bioenv.gu.se; 5 Biomar AS, 7010 Trondheim, Norway; sansh@biomar.com; 6 Department of Biology, Faculty of Science and Technology, Norwegian University of Science and Technology, 7491 Trondheim, Norway; rolf.e.olsen@ntnu.no

**Keywords:** oxidative status, Atlantic salmon, environmental stress, seasonal photoperiod, ecophysiology

## Abstract

Farmed Atlantic salmon reared under natural seasonal changes in sea-cages had an elevated consumption of antioxidants during spring. It is, however, unclear if this response was caused by the increase in day length, temperature, or both. The present study examined redox processes in Atlantic salmon that were reared in indoor tanks at constant temperature (9 °C) under a simulated natural photoperiod. The experiment lasted for 6 months, from vernal to autumnal equinoxes, with the associated increase and subsequent decrease in day length. We found that intracellular antioxidants were depleted, and there was an increase in malondialdehyde (MDA) levels in the liver and muscle of Atlantic salmon with increasing day length. Antioxidant enzyme activity in liver and muscle and their related gene profiles was also affected, with a distinct upregulation of genes involved in maintaining redox homeostasis, such as peroxiredoxins in the brain in April. This study also revealed a nuclear factor-erythroid 2-related factor 2 (Nrf2)-mediated oxidative stress response in muscle and liver, suggesting that fish integrate environmental signals through redox signaling pathways. Furthermore, growth and expression profiles implicated in growth hormone (GH) signaling and cell cycle regulation coincided with stress patterns. The results demonstrate that a change in photoperiod without the concomitant increase in temperature is sufficient to stimulate growth and change the tissue oxidative state in Atlantic salmon during spring and early summer. These findings provide new insights into redox regulation mechanisms underlying the response to the changing photoperiod, and highlight a link between oxidative status and physiological function.

## 1. Introduction

Most living organisms inhabit a highly dynamic environment characterized by daily and annual photoperiod/light rhythms [1]. Animals have evolved an internal system known as the circadian rhythm or other biological clocks with different periodicities, such as the circannual clock (annual cycle), to adapt to predictable changes in the environment [2]. The photoperiod serves as the primary entraining stimulus for the endogenous rhythm, providing cues for regulating physiology and behavior. In teleost fish, the photoperiod influences their life cycle from embryonic development [3] to sexual maturation [4] in adults. The photoperiod affects the growth of Atlantic salmon [5,6], and is considered a major cue for the parr-smolt transformation [7]. In the absence of photoperiod cues, the parr-smolt transformation is incomplete, as the osmoregulatory machinery and metabolism fail to meet the necessary requirements for salmon to adapt to the seawater environment [8]. Although the mechanisms underlying how light-sensitive structures mediate photoperiodic signals and regulate key physiological events in fish are not yet fully understood, it is suggested that light stimuli perceived by the retina and the pineal gland are transduced into hormonal or neural signals through the light–brain–pituitary axis [4,9].

Reactive oxygen species (ROS) are inevitably generated during aerobic and anaerobic metabolism. Excessive ROS accumulation causes damage to biomolecules. To prevent from oxidative stress, fish have developed an integrated antioxidant defense system that comprises both low- and high-molecular-mass antioxidants [10,11]. Low molecular mass antioxidants include water-soluble compounds such as ascorbic acid (Asc, vitamin C), and lipid-soluble ones such as α-tocopherol (α-TOH, Vitamin E). Vitamin C is considered a reducing agent, playing a significant role as an antioxidant, which can scavenge the ROS, and in turn, prevents cellular radical damage. Another antioxidant function is that vitamin C is hypothesized to recycle oxidized vitamin E [12]. Vitamin E competes with polyunsaturated fatty acids (PUFA) in donating a hydrogen atom to the lipid peroxyl radical, thereby breaking the chain of reactions involved in lipid auto-oxidation. High molecular mass antioxidants can be either specific or non-specific proteins. Specific proteins include antioxidant enzymes such as superoxide dismutases (tSod), glutathione peroxides (Gpxs), and catalase (Cat), which remove various ROS produced by free radical reactions. On the other hand, non-specific high-molecular-mass antioxidants such as metallothioneins bind to transition metal ions, providing protection against ROS-induced damage.

On the other hand, ROS, particularly hydrogen peroxide (H_2_O_2_), are widely recognized as crucial signaling messengers that coordinate a multitude of physiological functions [13]. These molecular species primarily trigger a reversible oxidative modification of cysteine thiols in regulatory proteins, influencing their protein structure and function and thereby cell signaling [14]. For example, with the increase in cellular oxidants, several cysteine residues of the Kelch-like ECH-associated protein 1 (Keap1) can be subject to oxidation. This oxidation leads to conformational change in Keap1, which loses its ability to ubiquitinate nuclear factor-erythroid 2-related factor 2 (Nrf2), allowing the Nrf2-induced transcription of numerous cytoprotective genes, including many from the antioxidants, thioredoxin (Txn), and GSH synthesis pathways [15]. Redox environments are regulated in cells, organelles, and subcellular compartments by various redox couples, of which the glutathione couple (reduced glutathione (GSH) and oxidized glutathione (GSSG)) is the most important [16]. 2GSH/GSSG is the major redox couple in cells and can be used as an indicator of the cellular redox environment [17]. Emerging evidence suggests that regulatory proteins involved in the cell cycle are subject to redox regulation [18,19]. The state of the redox environment is important for growth and development in mammals [20]. In line with this, growing evidence links the redox environment in fish to embryonic and larval development [21,22,23,24]. We recently found that implantation of the growth hormone (GH) regulates the expression of redox-related genes in Atlantic salmon [25]. This indicated that redox signaling may play an important role in the growth of fish.

Atlantic salmon reared in seawater in the spring are characterized by both increasing growth rates and accelerated tissue oxidative stress [26,27]. However, whether the increased oxidation was attributed to the seasonal change in photoperiod in Atlantic salmon is unclear. Photoperiod manipulation induces increased oxidative effects, which have been demonstrated in several fish species [28,29,30,31]. Therefore, the present study aims to investigate the effect of the seasonally changing photoperiod on redox metabolism and explore the potential associations with the growth of Atlantic salmon.

## 2. Materials and Methods

### 2.1. Fish and Experimental Design

**Figure 1:**
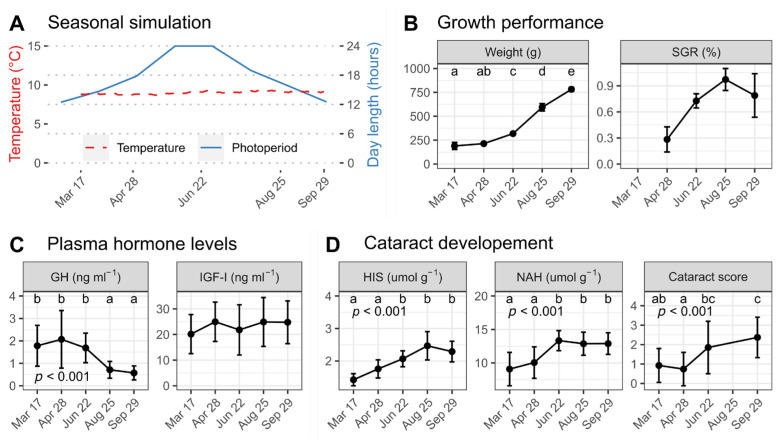
Seasonal effect of simulated natural photoperiod at constant temperature on growth, plasma hormone levels, and cataract development in Atlantic salmon. (**A**) Schematic of simulated natural photoperiod (blue line) and constant temperature (red dotted line) setting from March to September. Weight and specific growth rate (SGR) (**B**); plasma growth hormone (GH) and insulin-like growth factor I (IGF-I) levels (**C**); and the lens histidine (HIS), N-Acetyl histidine (NAH) levels, and cataract score (**D**) in Atlantic salmon sampled from 17 March to 29 September. Weight and SGR based on average values per tank, giving *n* = 3. GH, IGF-I, and cataract score were measured on 9 fish per tank (*n* = 27). HIS and NAH were measured on 3 fish per tank (*n* = 9). Data are means ± SD. Different letters indicate significant statistical differences between sampling points.

The experiments were carried out with Atlantic salmon of the AquaGen strain at the Institute of Marine Research (Matredal, 61° N, Western Norway). The salmon were produced as postsmolts in the autumn of 2019. On 23 October 2019, the Atlantic salmon were acclimatized to seawater and then exposed to a simulated natural photoperiod under constant temperature conditions of 9 °C. On 17 March 2020, a total of 300 Atlantic salmon with an average weight of approximately 196 g were randomly distributed into triplicate circular fiberglass tanks (1.5 × 1.5 × 0.45 m). The light phase was set once every month and then automatically increased (or decreased) gradually until the next month of the experiment (Figure 1A). During the trial, fish were fed ad libitum by automated feeders, with a basal diet containing 230 mg kg^−1^ α-tocopherol equivalents as tocopherol acetate and 440 mg kg^−1^ ascorbic acid equivalents as ascorbyl biphosphate. Formulation and major nutrients (analyzed) in the diet used here are given in Table 1.

### 2.2. Fish Sampling

Nine fish per tank were sampled on 17 March, 28 April, 22 June, 25 August, and 29 September. Fish were killed by an overdose of Finquel vet. (MSD Animal Health Norge AS; 0.3 g L^−1^), and the body weight (BW) was recorded. The cataract score of the fish lenses was determined using a Heine HSL 150 hand-held slit lamp (HEINE Optotechnik GmbH & Co. KG, Herrsching, Germany) under dark conditions. Each lens was scored on a scale of 0 to 4 based on its opacity, where 0 indicates no opacity and 4 indicates maximum opacity. The scores for both eyes were then summed, resulting in a total score ranging from 0 to 8 for each fish [32]. A blood sample was drawn from the caudal vein with a heparinized syringe and then centrifuged for 10 min at 3000× *g* at 4 °C. Plasma was removed and stored at −80 °C until radioimmunoassay for GH and IGF-I. The lens, liver, muscle, and brain were dissected and quickly frozen in liquid nitrogen. They were stored at −80 °C until analysis.

### 2.3. Radioimmunoassay

Plasma GH levels were measured using a specific double-antibody salmon GH radioimmunoassay (RIA), as outlined in [33] with modifications given in [34]. Plasma IGF-I levels were measured by double-antibody radioimmunoassay, following the procedures outlined in detail in [35].

### 2.4. Chemical Analyses of Feed and Tissues

L-histidine (HIS) and Na-Acetyl-L-histidine (NAH) levels in individual lenses were determined by reversed-phase HPLC (Waters Corporation, Milford, MA, USA) [36], as modified by Breck et al. [37]. Oxidized (GSSG) and reduced glutathione (GSH) were measured in 0.1 g samples using a commercial kit (Prod. No. GT40, Oxford Biomedical Research, Oxford, UK). Asc was measured in 0.5 g of pooled and homogenized samples of three fish per tank by HPLC [38], and α-TOH was analyzed according to the method of Hamre et al. [39]. Malondialdehyde (MDA) was analyzed according to the method of Hamre et al. [26].

### 2.5. Antioxidant Enzyme Activity

Activities of tSod, Gpx, Cat, and glutathione reductase (Gr) were measured in 150 mg of homogenized samples, using commercial kits (items 706002 (tSod), 703102 (Gpx), 703202 (Gr), 707002 (Cat); Cayman Chemical Co., Ann Arbor, MI, USA), as previously described [25].

### 2.6. RNA Extraction and RNA Sequencing

The total RNA of tissues was isolated on Biomek 4000 workstation by using Promega simplyRNA kit (AX2420, Madison, WI, USA). The RNA quantity was assessed by Nanodrop spectrophotometer (ND-1000, NanoDrop Technologies, Wilmington, DE, USA) and the quality was elevated by Agilent RNA 6000 Nano Kit in 2100 Bioanalyser (Agilent Technologies, Santa Clara, CA, USA). RNA-Seq library was prepared by the Illumina TruSeq Stranded mRNA library prep kit, according to the manufacturer’s protocol. A paired-end RNA-seq sequencing library was sequenced using the Illumina HiSeq 4000 system.

### 2.7. RNA-seq Preprocessing and Differentially Expression Analysis

The read quality checks, alignment, and differentially expressed genes (DEGs) were conducted as described by Yin et al. [25]. In brief, raw RNA-seq reads were trimmed and cleaned by TrimGalore! (Babraham Bioinformatics—Trim Galore!, version 0.6.5) and aligned to the reference genome of Atlantic salmon (ICSASG_v2; https://www.ncbi.nlm.nih.gov/assembly/GCF_000233375.1, accessed on 19 August 2022) by STAR [40] and quantified by featureCount [41]. The mRNA expression of genes chosen based on their importance within the redox system [21,22,23,25,42], oxidative stress response [43,44,45], and physiological processes including the GH–insulin-like growth factor (IGF) axis signaling [46,47], and cell cycle regulation [18,19] was studied in the liver, muscle, and brain (Supplementary Data S1). DEGs was defined by 10 pair-wise comparisons: (i) Apr vs. Mar, (ii) Jun vs. Mar, (iii) Aug vs. Mar, (iv) Sep vs. Mar, (v) Jun vs. Apr, (vi) Aug vs. Apr, (vii) Sep vs. Apr, (viii) Aug vs. June, (ix) Sep vs. Jun, and (x) Sep vs. Aug, by using the DESeq2 [48]. Genes were identified as DEGs when the adjusted *p* values were less than 0.05, and the absolute log fold changes (LFCs) were less than 1.2.

### 2.8. Gene Expression Analysis and Clustering

Read counts were normalized by the “Trimmed Mean of *M*-values” normalization (TMM) function provided by edgeR [49], and scaled by the minimum and maximum counts to values between 0 and 1. These normalized counts were formed into a count matrix and used as gene expression levels. Density-based spatial clustering of applications with noise (DBSCAN) [50] was used to identify similar expression patterns among a set of selected genes by utilizing their expression levels. The DBSCAN package (https://CRAN.R-project.org/package=dbscan, version 1.1-11, accessed on 16 September 2022) was used to identify DBSCAN clusters. The input values of DBSCAN were calculated as the differences in average TMM counts between 10 pairs of months. The pairs were (April, March), (June, March), (August, March), (September, March), (June, April), (August, April), (September, April), (August, June), (September, June), and (September, August). These differences in expression levels were then used as inputs to the DBSCAN package with the parameters (minimum points (“minpts”), epsilon (“eps”)): (12, 1.9) for liver, (10, 2) for muscle, and (10, 1.9) for brain.

### 2.9. Calculations

Specific growth rate (SGR, % per day) was calculated as:

(lnBW*_f_* − lnBW*_i_*) × 100/number of feeding days,

where BW*_i_* and BW*_f_* are the initial and final body weights (tank means), respectively.

Redox potential (Eh) of GSH/GSSG couple was calculated by the Nernst equation:

ΔEh = ΔE0′ − RT/nF ln([GSH]^2^/[GSSG]),

where R is the gas constant (R = 8.314 J K^−1^ mol^−1^), T is the temperature (in Kelvin), F is the Faraday constant (F = 9.6485 × 10^4^ C mol^−1^) and n is the number of electrons transferred. The GSH and GSSG levels are in moles and Eh in millivolts (mV). E0′ is the standard reduction potential assumed to be −240 mV at the environmental condition of 25 °C and pH 7 [17,51]. The redox potentials were calculated as the average of whole organs without consideration of the fact that they vary between different cell types and between organelles within the cells.

### 2.10. Statistical Analyses

All statistical analyses were performed using the free software environment R (http://cran.r-project.org/, R version 4.0.4, accessed on 15 February 2021). Data were statistically evaluated by nested one-way ANOVA (random effect = tank) followed by Tukey’s HSD post hoc test when significant differences were found. Shapiro–Wilk test and Levene’s test were used to examine the normality of distribution and homogeneity of the variances in the data, respectively (*p* > 0.05). The Kruskal–Wallis nonparametric test was used if the assumptions of normality or homogeneity were not met (*p* < 0.05). A principal component analysis (PCA) was performed (R: factoextra, version 4.0.5) in search for biological clusters involved in antioxidant status. Results are presented as means with standard deviation (mean ± SD) and differences were considered significant at *p* < 0.05. Potential outliers were analyzed (Grubbs’ test, alpha = 0.05) using GraphPad Prism, version 8 (GraphPad Software, La Jolla, CA, USA).

## 3. Results

### 3.1. Growth and Plasma Hormones

Weight increased from March to September and at a rapid rate from June to August, with the final weight (average 782 g) being around four-fold larger in September than in March (190 g). SGR increased significantly from April to a plateau in August-September (Figure 1B). The plasma GH levels were about 2 ng mL^−1^ in March, April, and June and then declined significantly to 0.5–0.7 ng mL^−1^ in August and September (Figure 1C). Plasma IGF-I levels remained stable around 20 ng mL^−1^ throughout the study (Figure 1C).

### 3.2. Lens Biochemicals and Cataract Development

The lens NAH level was low (around 9 µmol g^−1^) in March and April before significantly increasing to 13 µmol g^−1^ at the end of the trial (Figure 1D). The HIS content demonstrated an almost linear increase from March (1.4 µmol g^−1^) until reaching the highest level of 2.5 µmol g^−1^ in August (*p* < 0.001) when the content leveled off until the end of the trial in September. The cataract score was around 1 during March and April and increased to 1.9 in June and 2.4 in September (*p* < 0.001).

### 3.3. Asc, α-TOH, and MDA Levels

**Figure 2:**
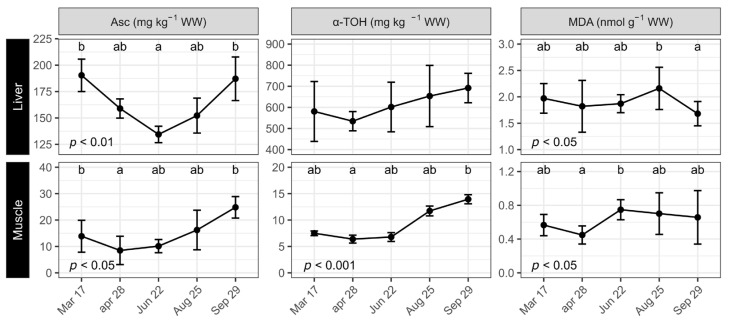
Asc, α-TOH, and malondialdehyde (MDA) in liver and muscle of Atlantic salmon under simulated natural photoperiod sampled from 17 March to 29 September. Vitamin C and vitamin E were measured on pooled samples of three fish per tank (*n* = 3 tanks). MDA was measured on 3 fish per tank (*n* = 9). Data are means ± SD. Different letters indicate significant statistical differences between sampling points.

Liver Asc levels were reduced from 190 mg kg^−1^ in March to 134 mg kg^−1^ in June, when the content started to increase again through to final sampling in September (187 mg kg^−1^; Figure 2). The α-TOH content was relatively stable at around 600 mg g^−1^ until April when there was a notable but nonsignificant increase towards the end of the trial (692 mg kg^−1^). Liver MDA remained constant at around 2 nmol g^−1^, peaking somewhat in August before being reduced in September to 1.68 nmol g^−1^ (*p* < 0.05).

Muscle Asc dropped significantly from 14 mg g^−1^ in March to 9 mg g^−1^ in April before increasing steadily to 25 mg g^−1^ in September (*p* < 0.05). Muscle α-TOH was stable at around 8 mg kg^−1^ until June when the content increased to 14 mg kg^−1^ by the end of the trial in September. The MDA content was relatively stable throughout, at around 0.7 mmol kg^−1^, except for an intermediate tendency to be reduced in April.

### 3.4. Glutathione Status

**Figure 3:**
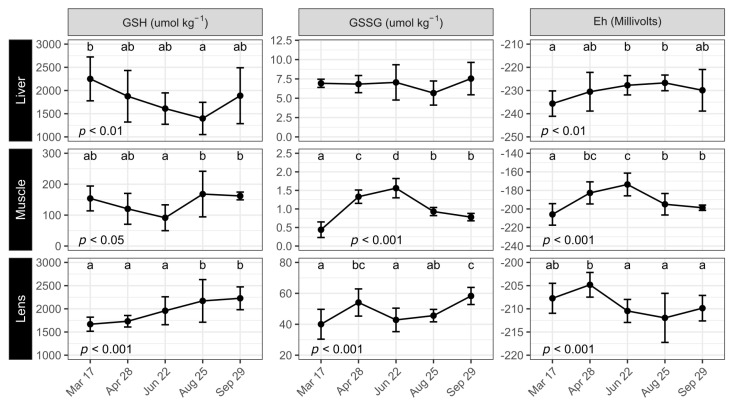
Reduced and oxidized glutathione (GSH and GSSG) and the redox potential (Eh) in liver, muscle, and lens of Atlantic salmon under simulated natural photoperiod from 17 March to 29 September. GSH, GSSG, and Eh were measured on 3 fish per tank (*n* = 9). Data are means ± SD. Different letters indicate significant statistical differences between sampling points.

Liver and muscle GSH levels decreased gradually from the beginning of the experiment until June for muscle and August for liver (*p* < 0.01, *p* < 0.05; Figure 3). It was also interesting to note that the liver GSSG level was rather stable throughout the trial (around 7 µmol kg^−1^) while it increased significantly in muscle from 0.4 µmol kg^−1^ in March to a maximum of 1.6 µmol kg^−1^ in June before falling again to 0.8 µmol kg^−1^ in September. The redox potential increased steadily in both liver (*p* < 0.01) and muscle (*p* < 0.001) from the start of the trial, peaking around June for both tissues. However, in liver, the level stabilized while in muscle it was reduced after reaching −199 mV in September. Lens GSH was around 1700 µmol kg^−1^ in March and April before increasing until reaching 2171 µmol kg^−1^ (*p* < 0.001) in August and levelling off until the end of the trial. The lens GSSG content varied somewhat over time, initially increasing from March to April (40 to 54 µmol kg^−1^), then dropping to 43 µmol kg^−1^ in June before slowly increasing to 58 at the end of the trial. Lens Eh was initially rather high (around −205 mV) in March and April before being reduced to around −210 mV from June to September (*p* < 0.001).

**Figure 4:**
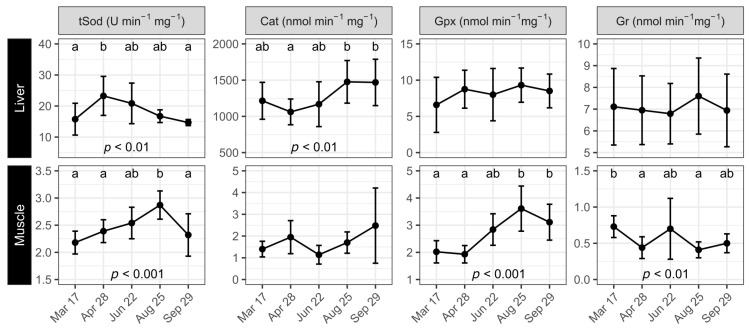
Changes in specific antioxidant enzyme activities in liver and muscle of Atlantic salmon under simulated natural photoperiod from 17 March to 29 September. Antioxidant enzyme activity was measured on 3 fish per tank (*n* = 9). Data are means ± SD. Different letters indicate significant statistical differences between sampling points.

The lowest level of liver tSod (around 16 U min^−1^ mg^−1^) was recorded in March and September (*p* < 0.01, Figure 4), after having peaked in April (23 U min^−1^ mg^−1^). The Cat level was reduced from March to April before increasing and leveling off at 1477 nmol min^−1^ mg^−1^ in August and September (*p* < 0.01). Gpx and Gr activities in liver were stable and did not change over the study period (*p* > 0.05). Muscle tSod levels rose from 2.2 U min^−1^ mg^−1^ in March to 2.9 U min^−1^ mg^−1^ in August, and subsequently fell again through September to 2.3 U min^−1^ mg^−1^ (*p* < 0.001). Muscle Cat did not change over the study but appeared to have a spike in September (*p* > 0.1). Muscle Gpx was stable between March and April at around 2 and then rose until reaching a peak level in August (3.6 nmol min^−1^ mg^−1^) that changed little by the end of the trial (*p* < 0.001). Muscle Gr varied somewhat and did not show any pattern of seasonality. The levels were higher in March and June (around 0.7 nmol min^−1^ mg^−1^), and lower (around 0.4 nmol min^−1^ mg^−1^) in April, August, and September (*p* < 0.01).

### 3.5. PCA Analyses

**Figure 5:**
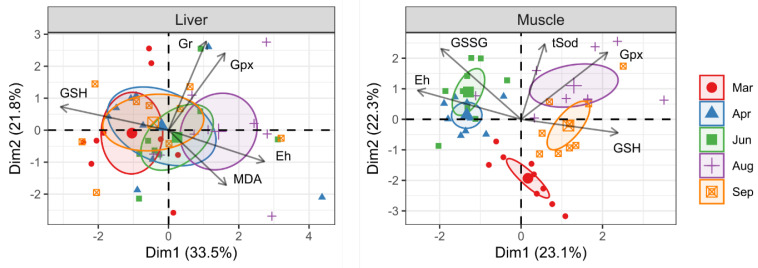
PCA biplots on antioxidants and oxidative markers measured in liver and muscle of Atlantic salmon under simulated natural photoperiod from 17 March to 29 September. The colored ellipse areas show the change in antioxidant capacity-related indexes over time. Arrows represent the 5 most contributing variables to the model (GSH, reduced glutathione; GSSG, oxidized glutathione; Eh, redox potential; Gr, glutathione reductase; Gpx; glutathione peroxidase; tSod, total superoxide dismutase).

The analysis of the antioxidant response revealed tissue-specific differences between the liver and muscle over the experimental period (Figure 5). The PCA biplots score plot demonstrated that the liver antioxidant status was clearly separated between March and August but not for April, June, and September. Two relevant components accounted for 55.3% of the overall variability, with PC1 explaining 33.5% of the total variance. PC1 was positively correlated with Eh and negatively correlated with GSH level. In contrast, the muscle antioxidant status was well grouped, and there was a clear separation of sampling points, except for April and June. PC1 and PC2 explained 23.1% and 22.3%, respectively, and both explained 45.4% of the variability in the muscle antioxidant status. The main contributing variables of PC1 were the high loading of Gpx activity and GSH level, which were positively correlated, and, on the other hand, Eh, which was negatively correlated.

### 3.6. Gene Expression

**Figure 6:**
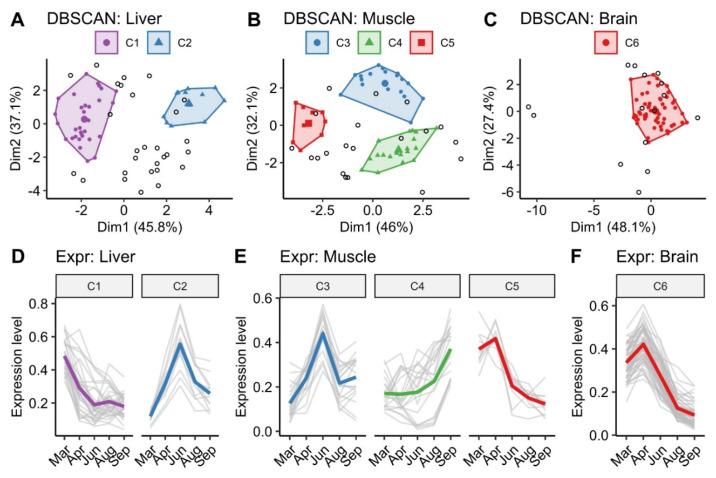
DEGs with threshold of log2(1.2) in liver, muscle, and brain clustered based on their change in expression in response to the change in day length. The white dots indicate genes without clustering. (**A**) Clusters in liver; (**B**) clusters in muscle; (**C**) cluster in brain; (**D**) gene expression patterns in the liver (C1 and C2); (**E**) gene expression patterns in the muscle (C3, C4, and C5); (**F**) gene expression patterns in the brain (C6). The colors represent similar clustering patterns: constant decrease (purple), peak in summer (blue), constant increase (green), and peak in spring (red).

Photoperiod manipulation had a significant effect on gene expression in the liver, muscle, and brain of Atlantic salmon in the present trial. DEGs with a log fold change (LFC) threshold (log2 (1.2)) are shown in Supplementary Data S2. Based on the DEGs (LFC = log2 (1.2)), DBSCAN analysis revealed a total of six clusters (designated C1–C6), of which two (C1 and C2), three (C3, C4, and C5), and one (C6) were in the liver, muscle, and brain, respectively (Figure 6A–C). Corresponding gene expression patterns for each tissue were shown in Figure 6D–F. Genes in cluster C2 and C3 had similar expression patterns with the seasonal change in day length, rising from March to a peak in June before declining. Conversely, genes in cluster C4 displayed the lowest expression levels in June, but they subsequently rose. Meanwhile, genes in clusters C1, C5, and C6 exhibited strong expression in the spring. Details of the gene families are listed in Table 2.

**Figure 7:**
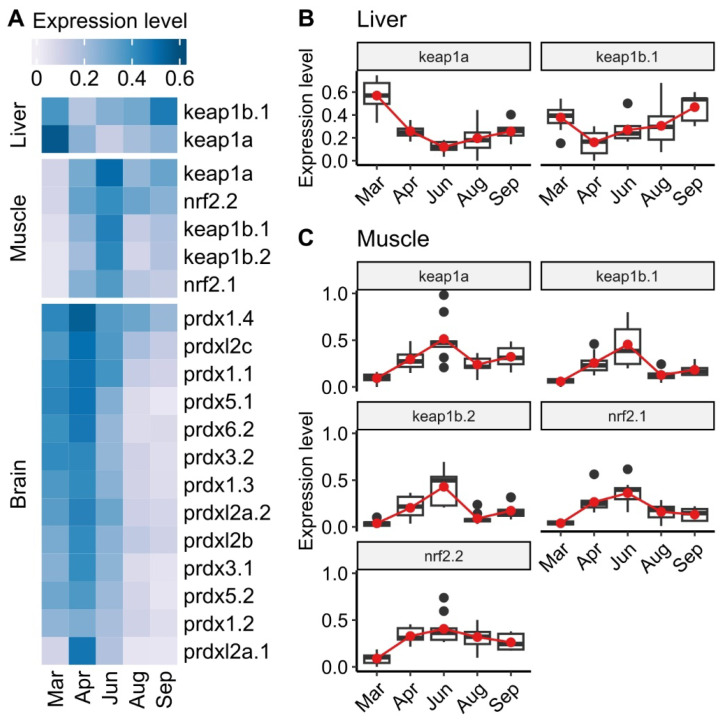
(**A**) Heatmap displaying mean normalized expression of the Keap1–Nrf2 pathway in the liver and muscle, and peroxiredoxin genes in the brain of Atlantic salmon (*n* = 9); dark blue and light blue indicate higher and lower gene expression level. (**B**,**C**) Significant gene expression of the Keap1–Nrf2 pathway in liver and muscle, respectively. Within the box plot, the horizontal line indicates the median value. The red line is drawn between the mean values in each group.

A heatmap of gene expressions involved in Keap1–Nrf2 pathway in the liver and muscle, and the peroxiredoxins in the brain, were shown in Figure 7A. In muscle, there were clear patterns of increased expression from March to June, followed by reductions until the end of the trial. This was particularly noticeable for transcription factors (*nrf2.1*, *nrf2.2 keap1a*, *keap1b.1*, and *keap1b.2*). The *keap1a* levels in the liver had the opposite trend reducing from March to June followed by a noticeable increase to September (Figure 7B,C). In the brain, over half (72%) of the analyzed genes coding for peroxiredoxins (Prdxs) were differentially expressed. In general, their expression increased from March to April before dropping to their lowest levels in September, and for many genes, below initial levels in March.

## 4. Discussion

This study demonstrates that the redox status of Atlantic salmon liver; muscle; and brain changes coincide with the seasonal change in photoperiod; and there are strong indications that this is linked to growth and cellular processes.

### 4.1. Depleted Tissue Antioxidants, Altered Antioxidant Enzymes, Genes, and Pathways of Redox Balance Indicate Oxidative Stress in the Spring and Early Summer

The change in day length led to significant changes in the oxidative status of Atlantic salmon tissues, with an apparent consumption of vitamin C and E. This is in line with Nordgarden et al. [27] and Hamre et al. [26]. Despite having sufficient dietary concentrations of Vitamin E, as indicated by previous studies [12,52], fish appear to develop Vitamin E deficiency due to their extraordinarily high consumption of muscle α-TOH. A previous study has found that the whole-body concentration of borderline Vitamin E deficient fish was between 6 and 12 mg kg^−1^ when dietary concentrations were 30–60 mg kg^−1^ [53]. In the present study, muscle α-TOH was below 10 mg kg^−1^. The high consumption of these antioxidant vitamins indicates that fish were exposed to increased oxidation, which was also reflected by the higher MDA, one of the final products of PUFA peroxidation in the cells. Therefore, the current study confirms the results of Hamre et al. [26]. Specifically, our results demonstrate that the change in photoperiod in indoor tanks with a constant temperature is sufficient to stimulate growth and induce changes in the tissue oxidative state.

GSH, an important endogenous antioxidant, is known to be synthesized when organisms are exposed to oxidative stress. In the present study, the expression of GSH synthetic enzymes was upregulated, but GSH was depleted during the period of increasing day length. The consumption of GSH was therefore higher than its production. This was reflected by a more oxidized redox potential in fish tissues. The redox potential is important in determining a diverse number of metabolic processes, including enzyme activity, signal transduction, and redox sensitive transcription factors [17,51,54]. It is suggested to be strictly regulated in healthy Atlantic salmon [55]. Therefore, the different redox status may explain some of the differences in gene expression levels and enzyme activities observed. The expression of many redox system genes in muscle, for example, genes clustered in C4 (*gpx2l.2*, *gstp.3*, *txnipl.1*, and *prdx4.2*), as well as stress response genes (*hsp60.2, hspa9, hsp90a.2*), mirrored changes in muscle GSSG levels and redox potential.

Although this work demonstrates that the seasonal change in photoperiod leads to changes in oxidative stress in Atlantic salmon tissues, the potential signaling pathway(s) involved in the regulation have remained unclear. We found that many genes important for mitochondrial metabolism and redox environment—*pgc-1a*, *ucp2*, *foxo3*, *pink*, *bnip3*, *nrf2*, *gpx1a*, *sod2*, *msrb2*, *glrx2*, *prdx3*, and *prdx5* [42,56,57,58,59,60]—were differentially regulated in tissues during the spring and early summer. Since mitochondria are central to energy metabolism [61] and are the primary source of intracellular ROS [62], increased mitochondrial metabolism may contribute to increased ROS production in the fish due to increased spring growth. Furthermore, transcription factors of the antioxidant response (*nrf2*, *keap1a*, and *keap1b*) were differentially regulated in the liver and muscle. The upregulation of Nrf2 or downregulation of Keap1 with the same oscillations of many cytoprotective target genes, such as those involved in GSH synthesis, suggests that increased day length induced an Nrf2-mediated oxidative stress response. This result coincides with earlier observations of the photoperiod response in gibel carp and hamster [30,63]. Heat shock proteins (HSPs) which function as molecular chaperones and are involved in protein folding, repair, refolding, or degradation of misfolded peptides, increase dramatically in response to cellular stress [64]. The increased expression of HSPs indicates protection against oxidative stress. Taken together, the increases in day length during spring resulted in increased oxidative stress, corresponding to lower vitamin C, E and oxidized redox potential in tissues. It can be speculated that following the redox regulation in the spring, which involved the induction of the Nrf2 pathway and the expression of several cytoprotective genes, the antioxidant vitamins and GSH recovered in the autumn. The oscillation is a possible mechanism for fish to cope with the changing environmental challenges for survival [65].

### 4.2. There Was a Distinct Gene Expression Profile Involved in Redox Control of the Brain in Response to Seasonal Change in Photoperiod

The brain utilizes redox signals for many functions [66]. However, it is prone to oxidative damage because of the high energy requirements and high levels of PUFA, which are susceptible to lipid peroxidation [67]. Excessive ROS in the brain is directly responsible for cell and tissue dysfunction [68]. It is therefore essential to maintain brain redox homeostasis. Notably, there was a distinct expression pattern in the brain, especially for genes involved in maintaining the redox balance. The upregulated expression of many genes encoding selenoproteins and the thiol oxidoreductase family in the present study suggests that increasing day length increases oxidative stress in the salmon brain, and that this change in gene expression protected it from oxidative damage. Studies have suggested that the brain is a good indicator of oxidative stress in aquatic organisms facing a plethora of environmental changes [69]. However, to the best of our knowledge, our study is the first to indicate that seasonally varying photoperiods directly influence salmon brain redox balance.

The peroxiredoxins are particularly interesting, as their expressions were upregulated with increasing day length. Peroxiredoxins are abundant intracellular H_2_O_2_-removing enzymes [70] and conserved markers of the circadian rhythm [71]. The relationship between circadian rhythms and the cellular redox system has attracted much attention [71,72,73], where oscillations of peroxiredoxins’ oxidation, H_2_O_2_, and redox status occur in parallel with the expression of clock genes in all aerobic organisms. This is supported by earlier observations in zebrafish, which imply that light utilizes H_2_O_2_ as the second messenger to influence endogenous circadian oscillations [74]. On the other hand, peroxiredoxins also have an emerging role as redox relay hubs, transmitting oxidizing equivalents from H_2_O_2_ to other target proteins [75]. It is therefore possible that the peroxiredoxin-thioredoxin system endows redox signal transduction in the neuronal system [58]. H_2_O_2_ is a major component in redox signaling given its relatively stable properties. The control of H_2_O_2_ gradients by several aquaporins (Aqp3, Aqp5, Aqp8, Aqp9, and Aqp11) allows further possibilities of redox signal transduction between organs, cells, and subcellular organelles [76], as shown for Aqp8 [77]. If increased day length-induced oxidations are mediated by circadian signaling, upregulated *prdxs* in the brain and differentially expressed *aqps* in tissues may play a role in redox signal transit. However, the effects of circadian signaling on salmonids’ redox system are poorly understood and need further research.

As in the brain, the retina is rich in PUFA, which increases its susceptibility to oxidative deterioration [78]. Oxidative stress is a prominent factor in the development of cataracts, and maintaining a balanced redox state is crucial for lens transparency [79]. HIS is taken up by the lens and converted to NAH, which has been linked to lens buffering [37] and osmolyte [80,81], as well as antioxidant effects [82]. In the present study, we found that fish developed cataracts, which is consistent with Hamre et al. [26]. We expected to find a parallel decrease for HIS and NAH as in a previous study [26], but on the contrary, their levels increased. These differences could be explained by variations in other environmental factors, for example temperature, which plays an important role in cataract development in salmon [83,84,85]. Correspondingly, the highest cataract score in the current study is also lower than that of salmon reared in sea cages [26]. The lens redox potential and GSSG spiked in the spring, which corresponded to the peak of genes in the brain. Earlier observations in mammalian cells and zebrafish suggest that light induces H_2_O_2_ production via photoreduction of flavin-containing oxidases [74,86]. Accordingly, our result suggests a redox crosstalk between the lens and brain, as photic information received by the photoreceptors of the retina and pineal organ is conveyed to the brain in fish [9]. However, whether and how fish integrate the photic information involved in redox-mediated signal transduction needs further study.

### 4.3. Redox Crosstalk with Growth and Cellular Processes Following the Seasonally Varying Photoperiod

Fish growth, expressed as SGR, from March to September in indoor tanks was comparable to earlier findings in sea cages [26]. In the current study, there is an inverse relationship between plasma GH levels and SGR, with the lowest plasma GH levels found during August–September when SGR is the highest. This is in agreement with previous studies, suggesting a rapid receptor binding and turn-over of hormone during periods of fast growth [7,87]. Together with some differentially expressed genes coding for growth hormone receptors (GHRs) and IGF binding proteins (IGFBPs), these results suggest that the photoperiod affects the GH-IGF-I system. Thus, the high SGR throughout the spring and summer in the current study may be attributed to activated GH-IGF-I signaling. The release of GH is regulated by factors of many kinds under hypothalamic regulation [47,88,89]. Some *ghr* and *igfbp* genes were expressed in a similar pattern to genes involved in the maintenance of redox balance during the spring, e.g., the C1 and C3 clusters in liver and muscle, respectively. In plants, hormones that regulate growth, development, and defense utilize ROS as second messengers [90]. Recently, we found that GH treatment has an oxidative effect on Atlantic salmon, resulting in increased oxidation and altering genes and pathways involved in redox regulation [25]. Consequently, the higher oxidation in the tissues of Atlantic salmon found here could be a result of GH signaling. However, some genes and pathways behaved differently, i.e., the NOX family, where some regulatory subunits were upregulated in the autumn when the fish had a lower plasma GH level. NOXs generate modest amounts of ROS that are important for intracellular signaling in nonphagocytic cells [91]. This discrepancy can be explained by the different signaling pathways that NOXs participate in, but the mechanism involved is still unknown.

In the present study, cell cycle and proliferation genes were highly expressed during the long day length period, suggesting photoperiod affected cellular processes such as mitosis and cell proliferation. It is known that continuous light causes an increase in proliferation and muscle fiber recruitment [92]. However, whether these mechanisms are active conditions of oxidative stress is unclear. The tight regulation of the cell cycle is achieved by the oscillation of cyclin and cyclin-dependent kinases (CDKs) activities, which are redox-dependent [18,19]. In Atlantic cod, the ontogenetic mRNA expression of *pcna*, *cdk2*, and *ccne2* exhibited a similar shift to *gclc* and redox status [22]. In keeping with the findings in this study, genes’ coding for cell cycle processes and redox balance shared similar expression patterns in the brain as well as in the muscle. This suggests a tight relationship between the seasonal photoperiod and cell cycle processes in patterning redox-related genes.

## 5. Conclusions

Increased oxidative stress in Atlantic salmon associated with the increase in day length during the spring and early summer manifests itself in the depletion of tissue antioxidants and an oxidized cellular environment, affecting many genes and pathways of antioxidant enzymes and the redox balance. These responses may be derived from the direct signaling of light, increased metabolism of mitochondrial ROS, or redox signaling through other pathways, such as GH or circadian clock signaling. The increased oxidation appears to stimulate fish growth and cellular functions, in line with previous findings in cells and different model organisms. These results therefore enlighten a potential interplay between physiological functions and oxidative responses in Atlantic salmon when exposed to seasonal variations in photoperiod.

## Figures and Tables

**Table 1:** Ingredient and chemical composition of the experimental diet.

	Diet
Ingredients (%)	
Fish Meal NA ^1^	22.5
Fish Meal SA ^2^	7.5
Soy protein concentrate ^3^	15.9
Maize gluten ^4^	10.0
Pea protein concentrate ^5^	8.4
Wheat meal ^6^	10.2
Fish oil ^7^	17.0
Rapeseed oil ^8^	6.5
Mono-sodium phosphate	0.83
Amino acid mix	0.77
Premix vitamins, minerals and others	0.96
Vitamin E (added)	0.05
Vitamin C (added)	0.13
**Analytical composition (% ww)**	
Protein	45
Lipid	26
Ash	6.3
Dry matter	94
**Analyzed micronutrients (mg kg^−1^ ww)**	
Ascorbic acid	440
a-TOH	230

^1^ FF-Skagen, Denmark. ^2^ Koster, Peru. ^3^ Nordsilmel, Brazil. ^4^ Nordsilmel, Ukraine. ^5^ Promill, China. ^6^ Hedegaard, Denmark. ^7^ FF-Skagen, Denmark. ^8^ Scanola, Denmark.

**Table 2:** DEGs with log2 (1.2) that belong to the different clusters.

Tissue	Clusters	Gene Family		
		Redox Balance	Oxidative Stress Response	GH Signaling and Cell Cycle
Liver	C1	*sepp*, *txnip.1*, *txnip.2*, *prdxl2a.2*, *prdx2b*,	*bcl2l1.1*, *hsp60.1*, *hsp60.2*, *hspa8.5*, *hspa8l*,	*ap-1*, *ghra*, *ghrb*, *ghr2*, *igf2b*,
		*g6pd.1*, *g6pd.2*, *ucp2.4*, *ucp2l*, *bnip3.3*,	*hspa4l.4*, *hsp90ab1*, *hsp90b*, *stip1l.1*, *stip1l.3*	*igfbp1a*, *igfbp1b1*
		*keap1a*, *nf-kb p65.2*, *ikba.3*, *aqp9*		
	C2	*gclc.1*	*bcl-2l.2*	*tp53.1*, *cdkn1a*, *pcna.1*, *pcna.2*, *e2f1*,
				*ccne2.1*, *ccne2.2*, *cdk1l*, *cdk2*, *cdk2l.1*,
Muscle	C3	*gpx2l.2*, *gstp.3*, *txnipl.1*, *prdx4.2*, *gclc.2*,	*hsp60.2*, *hspa9*, *hsp90a.2*	*igfbp5b1*, *igfbp3*, *pcna.1*, *pcna.2*
		*gclrl*, *gss*, *nrf2.1*, *nrf2.2*, *keap1a*,		
		*keap1b.1*, *keap1b.2*		
	C4	*gpx3*, *cat.1*, *sepp2*, *mt*, *msrb2.3*,	*hspb1l.2*, *bcl-2.2*, *bcl-2.1*	*ap-1l.2*
		*txn.3*, *prxdl2a.2*, *gclc.1*, *ucp2l*, *cybb*,		
		*ncf2.1*, *ncf4*, *cybbl.2*, *ikbα.4*, *maoa*		
	C5	*prdx3.2*, *txnrd2*, *pgc-1a.1*, *ikbab*, *foxo3l.2*	*hsp60.1*, *hspa8.1*, *hspa8.2*	*ghrb*, *ghr2*
Brain	C6	*sod1*, *sod1l*, *sod2*, *gpx1a*, *gpx2l.1*,	*baxl.3*, *bada*, *bad*, *hspb1.2*, *hspb1.3*,	*igfbp1a2*, *pcna.1*, *pcna.2*, *ccne2.2*,
		*gpx2l.2*, *gpx3l*, *gpx4*, *gpx4a*, *sepp*,	*hspb1l.1*, *hpbp1.1*	*cdk2*, *cdk2l.1*
		*gstp.1*, *gstp.3*, *mt*, *mt2*, *msra*,		
		*msrb1a*, *msrb1l.1*, *msrb2.2*, *msrb2.3*, *gclc.2*,		
		*gclr*, *gclrl*, *ucp2.4*, *pink*, *bnip3.1*,		
		*bnip3l.2*, *txn.2*, *txn.3*, *txnl4a.2*, *txnl4b*,		
		*glrx1.2*, *glrx2*, *prdx1.1*, *prdx1.2*, *prdx1.3*,		
		*prdx1.4*, *prdxl2a.2*, *prdxl2b*, *prdxl2c*, *prdx3.1*,		
		*prdx3.2*, *prdx5.1*, *prdx5.2*, *prdx6.2*		

## Data Availability

Results used in this publication will be shared upon reasonable request addressed to the corresponding author.

## Supplementary Materials

The following supporting information can be downloaded at: https://www.mdpi.com/article/10.3390/antiox12081546/s1, Supplementary Data S1: 336 genes involved in redox system, oxidative stress response, and physiological processes; Supplementary Data S2: Differentially expressed genes with a log fold change threshold (log2 (1.2)).
